# Elucidating Protein Involvement in the Stabilization of the Biogenic Silver Nanoparticles

**DOI:** 10.1186/s11671-016-1538-y

**Published:** 2016-06-29

**Authors:** Daniela Ballottin, Stephanie Fulaz, Michele L. Souza, Paola Corio, Alexandre G. Rodrigues, Ana O. Souza, Priscyla M. Gaspari, Alexandre F. Gomes, Fábio Gozzo, Ljubica Tasic

**Affiliations:** Laboratório de Química Biológica, Instituto de Química, Universidade Estadual de Campinas, Campinas, SP Brazil; NanoBioss, SisNano, Universidade Estadual de Campinas, Campinas, SP Brazil; Departamento de Química Fundamental, Instituto de Química, Universidade de São Paulo, São Paulo, SP Brazil; Instituto de Ciências Exatas, Universidade Federal Fluminense, Volta Redonda, RJ Brazil; Laboratório de Bioquímica e Biofísica, Instituto Butantan, São Paulo, SP Brazil; Laboratório de Nanobiotecnologia, Faculdade de Ciências Farmacêuticas, Universidade de São Paulo, Riberão Preto, SP Brazil; Laboratório Dalton, Instituto de Química, Universidade Estadual de Campinas, Campinas, SP Brazil

**Keywords:** Biogenic silver nanoparticles (AgNPs), Capping proteins, *Aspergillus tubingensis*

## Abstract

Silver nanoparticles (AgNPs) have been broadly used as antibacterial and antiviral agents. Further, interests for green AgNP synthesis have increased in recent years and several results for AgNP biological synthesis have been reported using bacteria, fungi and plant extracts. The understanding of the role and nature of fungal proteins, their interaction with AgNPs and the subsequent stabilization of nanosilver is yet to be deeply investigated. Therefore, in an attempt to better understand biogenic AgNP stabilization with the extracellular fungal proteins and to describe these supramolecular interactions between proteins and silver nanoparticles, AgNPs, produced extracellularly by *Aspergillus tubingensis*—isolated as an endophytic fungus from *Rizophora mangle—*were characterized in order to study their physical characteristics, identify the involved proteins, and shed light into the interactions among protein-NPs by several techniques. AgNPs of around 35 nm in diameter as measured by TEM and a positive zeta potential of +8.48 mV were obtained. These AgNPs exhibited a surface plasmon resonance (SPR) band at 440 nm, indicating the nanoparticles formation, and another band at 280 nm, attributed to the electronic excitations in tryptophan, tyrosine, and/or phenylalanine residues in fungal proteins. Fungal proteins were covalently bounded to the AgNPs, mainly through S–Ag bonds due to cysteine residues (HS–) and with few N–Ag bonds from H_2_N– groups, as verified by Raman spectroscopy. Observed supramolecular interactions also occur by electrostatic and other protein–protein interactions. Furthermore, proteins that remain free on AgNP surface may perform hydrogen bonds with other proteins or water increasing thus the capping layer around the AgNPs and consequently expanding the hydrodynamic diameter of the particles (~264 nm, measured by DLS). FTIR results enabled us to state that proteins adsorbed to the AgNPs did not suffer relevant secondary structure alteration upon their physical interaction with the AgNPs or when covalently bonded to them. Eight proteins in the AgNP dispersion were identified by mass spectrometry analyses. All these proteins are involved in metabolic pathways of the fungus and are important for carbon, phosphorous and nitrogen uptake, and for the fungal growth. Thereby, important proteins for fungi are also involved in the formation and stabilization of the biogenic AgNPs.

**Electronic supplementary material:**

The online version of this article (doi:10.1186/s11671-016-1538-y) contains supplementary material, which is available to authorized users.

## Background

Nanotechnology has attracted the attention of researchers worldwide because of the unique properties of nanomaterials. Countless applications have been studied in different fields, such as medicine [1, 2], material science [3], microelectronics [4], energy storing [5], and biomedical devices [6].

Silver nanoparticles (AgNPs) have been largely employed in antibacterial and antiviral applications [7–16]. They present antibacterial and antimicrobial activity against Gram-negative and Gram-positive bacteria and some viruses as well [17–19]. Silver ions attack several targets in the bacteria making the development of resistance difficult [20]. The enormous surface area of nanoparticles improves its penetrability into the cell, enhancing their antimicrobial action [21].

AgNPs can be produced by chemical [22, 23], physical or biological routes [24, 25]. Biological synthesis uses clean routes, without producing toxic residues. AgNP biosynthesis can be performed using bacteria [7, 26], fungi [27–30], yeasts [31], plant extracts [32, 33], cyanobacteria [34], algae [35, 36], and actinomycetes [37]. This synthesis can be extra- or intra-cellular [38–41].

Fungi are easy microorganisms to manipulate as they grow in mycelial form; they are more resistant facing adverse conditions and provide a cost-effective large-scale production [42]. For these reasons, fungi appear to be interesting microorganisms for the green synthesis of silver nanoparticles. Fungus *Aspergillus tubingensis* is part of the black Aspergilli as well as *A. niger*, *A. carbonarius*, and *A. aculeatus* [24, 41–46] that grows on plant material. Many species of *Aspergillus* section *nigri* exhibit important biochemical differences in secretome [47–49]. *A. tubingensis*, used in this instance, was isolated as an endophytic fungus from *Rizophora mangle* [28]*.* Similar to other fungi, *A. tubingensis* is unable to import polymeric compounds into the cell and relies on enzymatic degradation to produce monomers or oligomers from different plant polymers among which polysaccharides are the major constituents [50, 51]. Due to structural differences in the plant polysaccharides, their effective degradation depends on an efficient system that regulates the production and secretion of different enzyme cocktails.

*A. tubingensis* is normally grown in a rich medium, such as potato dextrose agar (PDA), removed from it and washed with clean and distilled water originating the fungal filtrate (FF), rich in proteins and fungal metabolites. Then, Ag(I) aqueous solution is added into the FF where redox reactions occur and AgNPs are formed [51, 52]. Although various investigations have reported the mechanism of production of AgNPs obtained through this extracellular synthesis using different biological agents [33, 38–40], little is yet known about the role and nature of fungal proteins and also about their interactions with AgNPs and the subsequent stabilization of the as-produced nanosilver [51–55].

Interactions between nanosilver and proteins lead to AgNP stabilization and the formation of nanoparticle-biomolecular-capped structures [56–58] that could be monitored by different techniques. These biophysical and biochemical interactions occur through covalent bonds and electrostatic interactions [59, 60]. Silver nanoparticles can be complexed with the thiol HS– (Cys) or amine H_2_N– groups [61–63] of the proteins and through electrostatic interactions [64] that have less impact on protein conformation and function. Sometimes, proteins covalently bound to AgNPs attract other proteins in order to form protein–protein-specific or nonspecific interactions that are an important part of the nanosilver-protein multilayer.

In an attempt to better understand biogenic AgNP stabilization with extracellular fungal proteins and to define these supramolecular interactions, we have chosen biogenic nanosilver with positive zeta potential. To the best of our knowledge, the present study is the first to report such data on covalently bound proteins to bionanosilver (AgNPs), synthesized by *A. tubingensis*. Biogenic AgNPs, of well-defined size and distinct morphology, are formed through the reduction of an aqueous solution of Ag(I) by a fungal filtrate.

Although the involvement of proteins in the reduction of the Ag(I) ions and the stabilization of a newly formed AgNPs has been described [23, 28, 64, 65], data about the way these proteins act are scarce. To fill the gap, the present study was devised in order to identify the proteins that promote the formation of AgNPs and those involved in the stabilization of the same nanomaterials.

## Methods

All chemicals used in this study were purchased from Sigma-Aldrich (St. Louis, MO, USA) and used without further purification unless otherwise stated.

Fungal strain of *A. tubingensis* (AY876924) was provided by I. S. Melo (Embrapa/CNPMA, Brazil) and is part of the culture collection of the “Embrapa Recursos Genéticos e Biotecnologia (CENARGEN)” in the “Collection of Microorganisms for Biocontrol of Plant Pathogens and Weeds” (http://mwpin004.cenargen.embrapa.br/jrgnweb/jmcohtml/jmcoconsulta-externa.jsp?idcol=11) under the number CEN1065.

### Silver Nanoparticle Synthesis

The endophytic fungi *A. tubingensis* was cultivated in potato dextrose agar medium (PDA) at 28 °C for 7 days. Afterwards, the fungal colonies were transferred to tubes containing 5 mL of saline solution (9 % NaCl). The obtained suspension was added to 150 mL of potato dextrose broth (PDB) in a 1-L Erlenmeyer flask and incubated in an orbital shaker (Marconi MA420, Brazil) at 25 °C and 150 rpm for 72 h. After this period, the biomass was filtered using a polypropylene membrane and washed with sterile water. After incubation with sterile water at 25 °C and 150 rpm for 72 h, the biomass was removed and the fungal filtrate (FF) was obtained using a cellulose acetate membrane of 0.22 μm.

For AgNP synthesis, 1 mL of AgNO_3_ solution (0.1 mol L^-1^), previously filtered through a cellulose regenerated membrane (0.22 μm), was added to 99 mL of the FF to reach a final concentration of 1 mmol L^−1^. The flask was kept at 25 °C and protected in dark for 96 h. The formation of AgNPs was monitored using a UV-Vis spectrophotometer (Agilent 8453). Control (FF without any silver ions) was used as blank. The average size (z-average) of AgNPs was measured by dynamic light scattering (DLS) (Nano ZS Zetasizer, Malvern Instruments Corp, UK) at 25 °C in polystyrene cuvettes with a path length of 10 mm. The zeta potential was measured in capillary cells with a path length of 10 mm, using the same instrument. The samples were diluted with 0.1 mmol L^−1^ NaCl before the analysis.

### Characterization of the Proteins Capping the AgNPs

*FTIR spectroscopy* measurements were carried out from KBr tablets of two samples, AgNPs and FF, and were recorded in an ABB Bomem (MB series, USA) instrument with a resolution of 4.0 cm^−1^ and in an interval from 4000 to 400 cm^−1^.

*Raman spectroscopy* measurements were implemented at the Instituto de Química, Universidade de São Paulo and recorded in a Renishaw InVia Reflex equipment coupled to a DM2500M Leica microscope using 632.8 and 785 nm lasers at 3 mW and 30 mW, respectively. Fifty-second accumulation in a total of three scans to each sample between 100 and 1800 cm^−1^ range were obtained, at 4 cm^−1^ resolution. All samples were analyzed in suspension and solid KCl was added in order to promote aggregation; however, no visual change was noticed.

*LC-MS/MS analysis* were performed at Laboratório Dalton, Instituto de Química, Universidade Estadual de Campinas using a nanoACQUITY chromatograph with a UPLC (Waters) coupled to a Synapt HDMS spectrometer (Waters) with QTOF geometry equipped with a nanoESI source operating in the acquisition-dependent data mode (ADD).

After being quantified by the Bradford method [66], proteins from the FF and linked to the AgNPs were analyzed by LC-MS/MS according to a method based on denaturation followed by digestion using the trypsin enzyme (Sequencing Grade Modified Trypsin, Promega), desalting and concentration. The resulting solutions were centrifuged (10 min at 17,000×*g*) and the supernatant was transferred into appropriate vials. Then, the samples were injected into the UPLC system, first passing through the precolumn (Waters Symmetry C18, 20 mm × 180 μm, particles 5 μm), being desalted during 3 min with a flow of 5.0 μL min^−1^ with 97:3 water/acetonitrile with 0.1 % formic acid (v/v) and, afterwards, they were transferred to the analytical column (Waters C18 BEH130, 100 mm ID × 100 μm, particles of 1.7 μm). Finally, the samples were eluted with a flow rate of 1.0 μL min^−1^ by varying the gradient of mobile phases with a gradient of buffer A (water/formic acid 0.1 %, v/v) and B (acetonitrile/formic acid 0.1 %, v/v) at the rates of 97:3, 70:30, 20:80, 20:80, 97:3, and 97:3 at 0, 40, 50, 55, 56, and 60 min, respectively. The identification of the peptides was done using the online version of the Waters software with a mass spectrometer (Synapt HDMS-Waters) configured to operate in dependent acquisition data (ADD) mode containing a function MS full-scan (*m/z* 200–2000), a three function fragment ion spectrum (MS/MS, m/z 50 to 50 units over the m/z of the precursor) and a function of external standard calibration (lock-mass, m/z 200–2000). All spectra were acquired at a rate of 1 Hz. The other parameters were capillary voltage of 3.0 kV, cone voltage of 30 V, source temperature of 100 °C Gas Flow nanoESI 0.5 L h^−1^, collision energies of 6:04 eV and a 1700-V detector. The acquisition of raw data was performed with ProteinLynx Global Server v.2.2 software (Waters). Data treatments for the deconvolutions of raw spectra were performed with Transform software (Micromass, UK). MASCOT v.2.2 system (Matrix Science Ltd. http://www.matrixscience.com). Data banks were searched in order to identify the fungal proteins.

## Results and Discussion

**Fig. 1:**
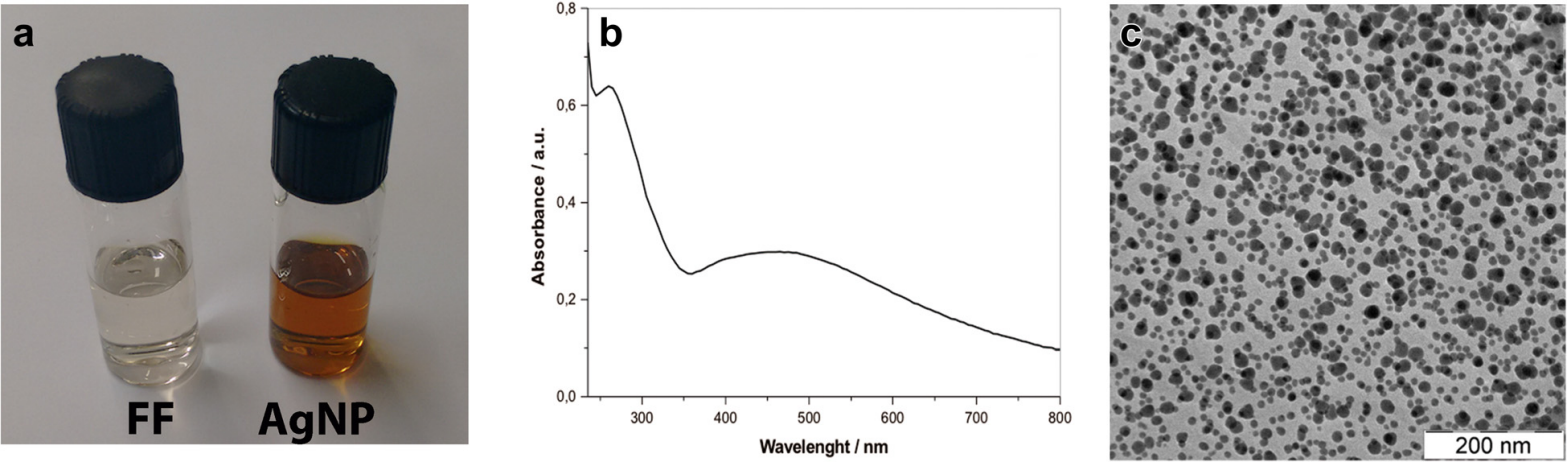
**a** Image of the fungal filtrate and the AgNP suspension. **b** UV-Vis spectra obtained for AgNP suspension using FF as blank. **c** Electronic Transmission micrograph showing the AgNPs

Biogenic AgNP formation through a fungal-based extracellular synthesis is a known, efficient, green, and relatively fast way for AgNP production [28, 58, 61, 62, 65, 67, 68] as this process takes a few days to complete (Fig. 1a). Herein, the biogenic synthesis was monitored by UV-Vis spectroscopy (Fig. 1b). The formation of AgNPs was completed within 72 h after the FF was challenged with AgNO_3_, in good agreement with what was previously reported [28]. The UV-Vis spectrum displays two main bands, an SPR band at 440 nm, characteristic of the AgNP presence, and an additional band at 280 nm, which could be attributed to the aromatic amino acids of the capping proteins [69]. It is well-known that the absorption band in this region arises due to the electronic excitations in tryptophan, tyrosine and/or phenylalanine residues in fungal proteins [69–71]. These results confirm the AgNPs formation and the presence of fungal proteins.

Silver nanoparticles were characterized, and their average diameter and zeta potential were evaluated. In DLS analysis, these AgNPs showed a hydrodynamic diameter of 264.9 ± 3.2 nm and relatively low polydispersity (0.32) (data not shown herein, already presented in [28]). Their zeta potential was positive with a value of + 8.48 ± 0.45 mV which could be indicative of low-charged surfaces and, consequently, unstable AgNPs [72], contrary to what was observed during a 6-month period. The high AgNPs stability might be attributed to the fungal protein-capping around the particles what confers them steric stability. The average diameter measured by TEM was 35 ± 10 nm (Fig. 1b and other data shown previously [28]). This value is smaller when compared to that measured by DLS, because in the latter technique the hydrodynamic diameter (particles and stabilization protein-capping) is taken into account [28], on the other hand, TEM allows the measurement of the AgNP diameter without the surrounding capping layers. Once again, strong evidence for fungal proteins linked to the silver nanoparticles was obtained.

**Fig. 2:**
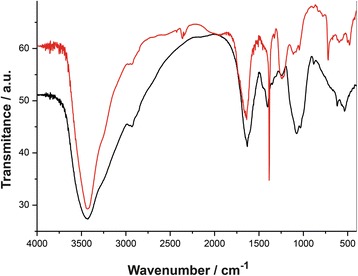
FTIR spectra of the fungal filtrate (*black*) and AgNPs (*red*) carried out in KBr tablets

Protein adsorption on the surface of biogenic AgNPs was also confirmed by FTIR spectroscopy (Fig. 2). For example, the peptide bond exhibits characteristic bands denominated amide A, B, I-VII. The Fermi resonance that occurs among the first overtone of amide II and the N–H stretching vibration create the bands amide A (about 3500 cm^−1^) and amide B (about 3100 cm^−1^) [72–76]. The band in 1600–1700 cm^−1^ named amide I is related with the C = O stretching vibration from the backbone conformation [72]. The amide II band arises from the N–H bending vibration and from the C–N stretching vibration [73] and is conformational sensitive. The complex bands Amide III and IV originates from a mixture of several coordinate displacements [77]. The symmetric and asymmetric vibrations of the C–H groups result in bands at 2920–2950 cm^−1^, respectively [78], while bands at 1620 to 1650 cm^−1^ are attributed to –C(O)– of peptide bonds and/or –NH_2_ groups and those at 1380–1030 cm^−1^ to C-N bonds [74, 75].

According to the FTIR results the proteins on AgNP surface did not undergo relevant secondary structure alteration along with their interaction with AgNPs, nor when covalently bonded to them as reported in other published data [50, 51]. The interaction between the proteins and AgNPs might be covalent bound to the amino groups, cysteine residues, and/or electrostatic interactions via carboxyl groups.

**Fig. 3:**
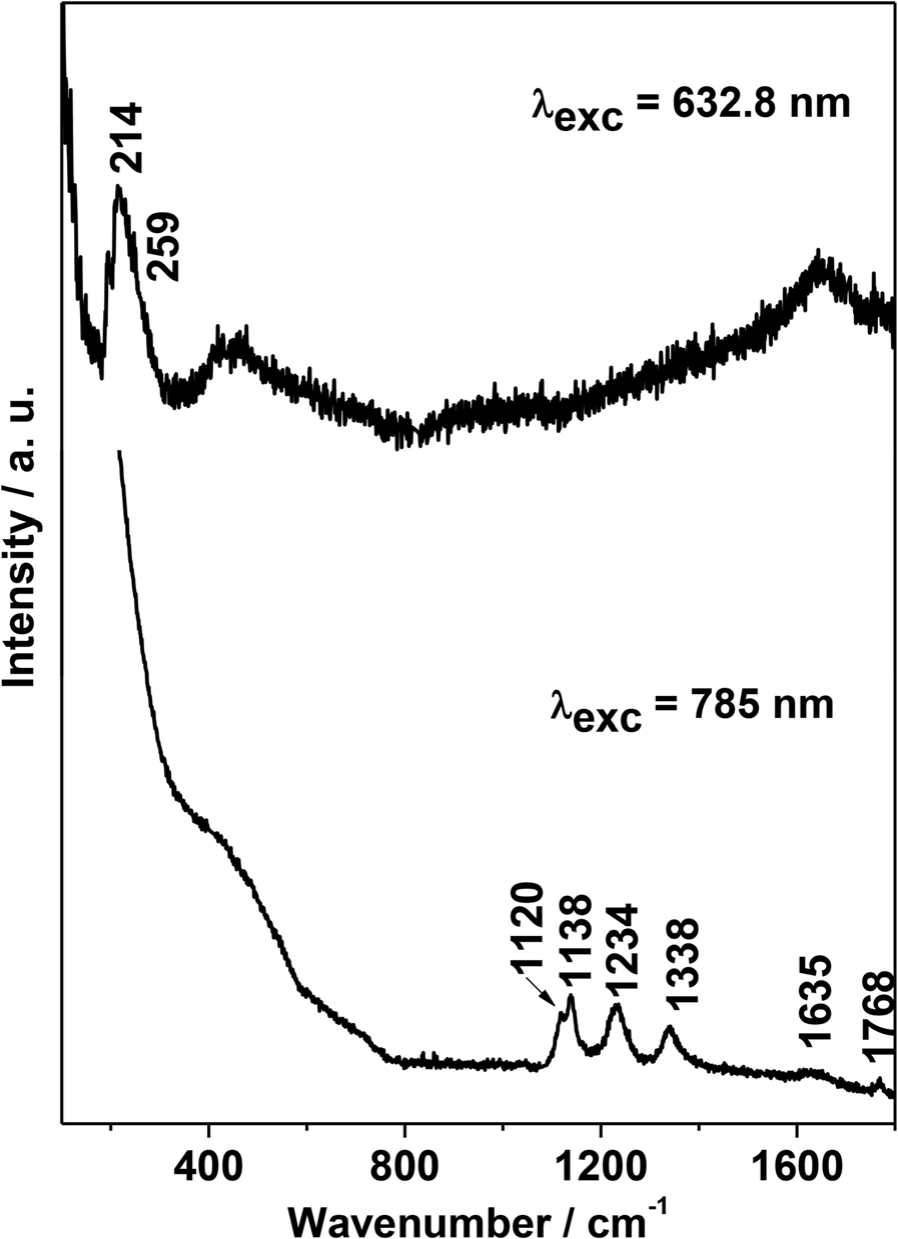
Raman spectra of AgNPs recorded with laser excitations of 632.8 nm and 785 nm. The main wavenumbers discussed further in text are pointed

The Raman spectra (Fig. 3) indicate the presence of protein-capping at the surface of the investigated AgNPs [77, 79, 80], confirming the DLS results for the hydrodynamic diameter. Moreover, Raman spectroscopy enable observe if the protein binding to the surface occurs via free amino groups or through cysteine residues. The spectrum excited at 632.8 nm presents little vibrational information about the molecules at the AgNP surface. The broad band at around 214 cm^−1^ can be assigned to an overlap between the Ag–Cl vibration (given the presence of Cl–) and an Ag–S vibration suggesting an interaction between superficial Ag and the cysteine (HS–) group of the capping proteins. When the samples were excited at 785 nm, strong bands assigned to the adsorbed proteins are observed at 1338 and 1768 cm^−1^, assigned to the amide III and amide I modes, respectively, as already discussed in the FTIR results above. Bands at 1120 and 1138 cm^−1^ are assigned to NCH stretching and CCH bending modes, respectively, and 1234 cm^−1^ to vibrations in antiparallel β-sheet in the protein structure [81]. A broad and weak band related to the amide II mode is present at approximately 1635 cm^−1^, which was expected to be at lower frequencies (below 1600 cm^-1^). The observed blue shift is associated to a response of the protein bonding to AgNPs, increasing the vibrational frequencies of the free amine II mode. On the other hand, it was expected to detect HCS bending between 800 and 900 cm^−1^. However, such peak was not present in any of the obtained spectra reinforcing that the binding of protein to the AgNP surface occurred mainly through the –SH groups. In such case, the amino group remains free and may perform hydrogen bonds with other proteins or water, contributing hence to the large hydrodynamic radius and the low charge surface of these NP. Therefore, proteins detected in AgNPs are covalently bound to the silver through S–Ag bonds, principally, and with some adhered proteins via electrostatic or other protein–protein interactions.

**Fig. 4:**
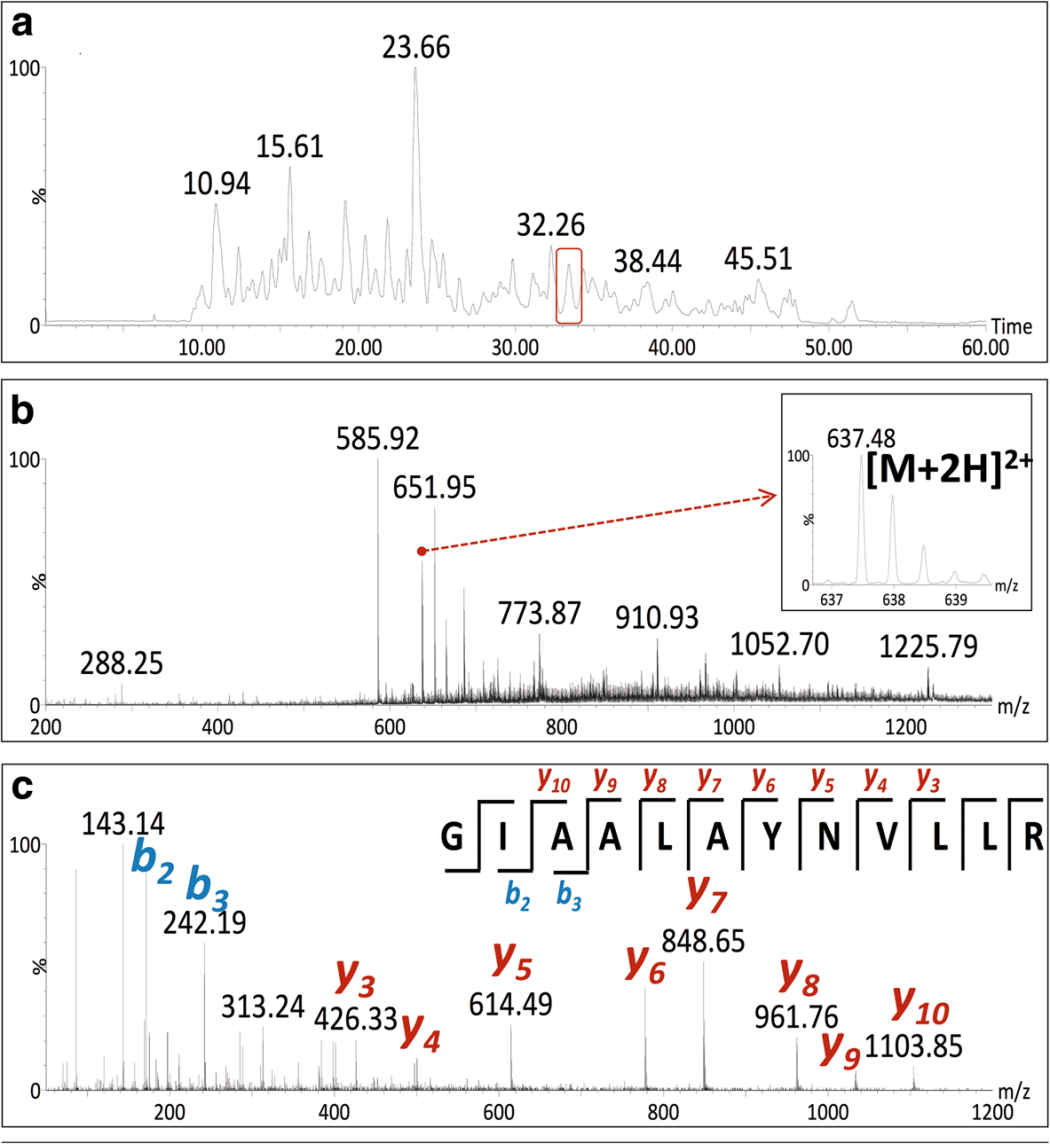
Illustration of the data obtained in performed MS/MS analysis. **a** The chromatogram is showing the eluation times for the AgNPs trypsin-hydrolyzed sample where sample’s peptides are given from 0 to 50 min; the peptide (*red box*) at 32.26 min was selected for posterior identification in MS. **b** Mass spectrum that corresponds to the peptide from 32.26 min (*red box* in **a**). **c** MS/MS data and procedure followed for the identification of the peptide sequence for the peptide from 32.26 min (*red box* in **a**)

The protein identification in the dispersion of AgNPs was performed starting from the protein tryptic lysis followed by LC-MS/MS analysis [82–88]. An illustration of the LC-MS/MS results obtained for proteins capping AgNPs is shown in Fig. 4 and all identified proteins are shown in Additional file 1: Table S1. The most intense signals in the chromatograms of peptides were selected for further fragmentation and, after obtaining their MS spectra, three to five most intense m/z ions were fragmented in MS/MS spectra allowing us to associate an amino acid sequence for a fragmentation pattern, as exemplified for one of the identified peptides (Fig. 4).

Mass spectrometry analyses enabled the identification of eight (8) proteins in the AgNPs dispersion and these are presented in Additional file 1: Table S1. All of them, secreted by *A. tubingensis*, display low isoelectric points, ranging from 4.0 to 5.1, characteristic for acidic proteins. Their molecular masses varied from 39 to 65.5 kDa.

*A. tubingensis* was grown in broth whose pH was 6.5 to 6.8 and, therefore, the fungus extracellular proteins should exhibit negative charge due to the deprotonation, which could increase the zeta potential of the synthetized AgNPs. Nevertheless, the positive zeta potential of approximately 8 mV, which should be indicative of low-charged surfaces, is probably a consequence of these protein-capping deprotonation. Some published data on chemical AgNPs and protein interactions also report similar observations [50].

**Fig. 5:**
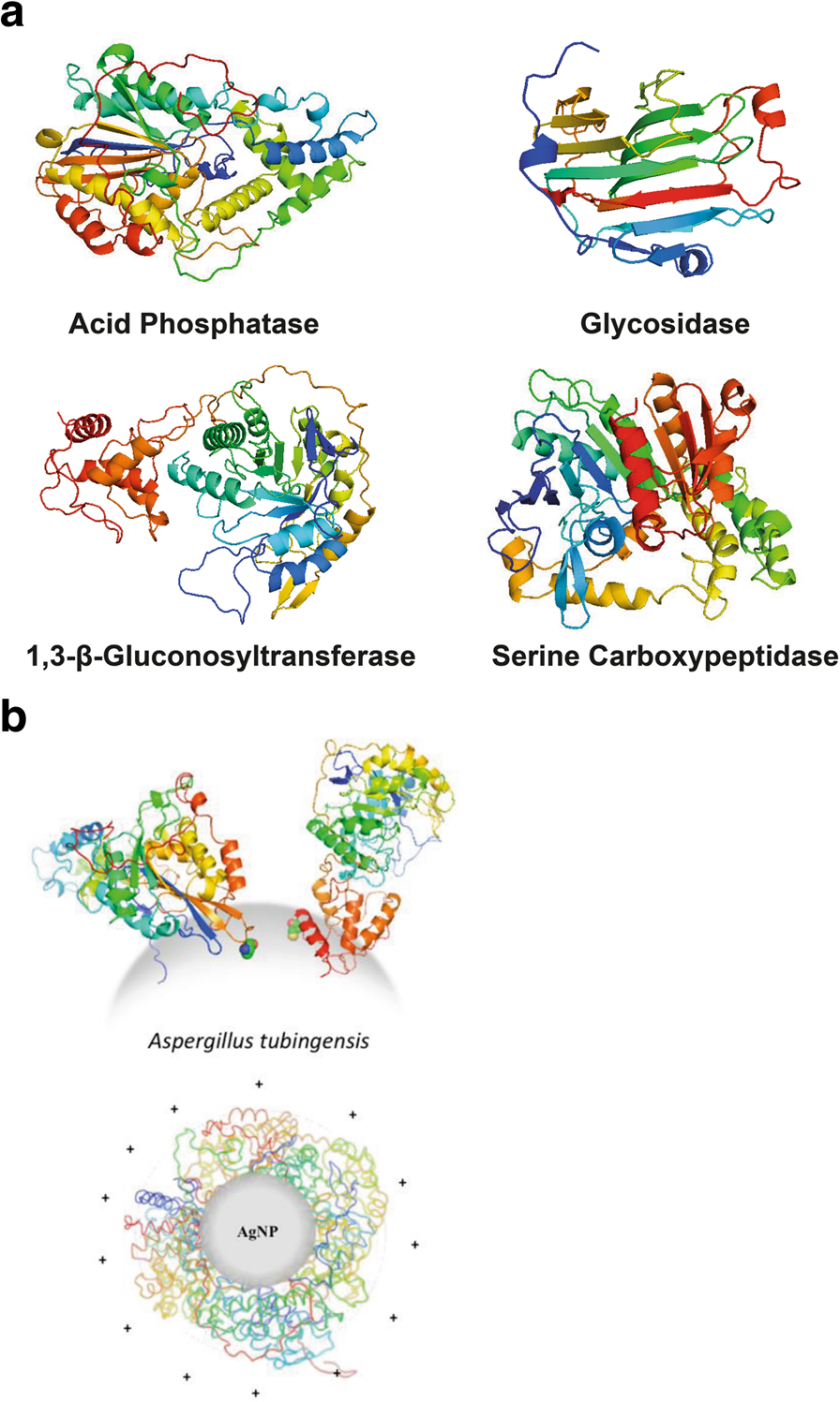
**a** Illustration of the 3D structures (in *ribbon*) of the most important biogenic AgNP proteins identified using MS/MS in biogenic AgNP characterization. **b** Representation of the proteins around the AgNPs

Among identified proteins, we have found glycoamilase (1,4-α-d-glucanglucohydrolase, EC 3.2.1.3), acid phosphatase (EC 3.1.3.2), serine carboxipeptidase (EC 3.4.21.26), and glucanosyltransferase (EC 2.4) that are illustrated in Fig. 5. All these proteins are involved in metabolic pathways of the fungi and belong to hydrolases [56, 89–93], important for carbon, phosphorous, and nitrogen uptake, respectively, and for the fungal growth. Furthermore, all identified hypothetic proteins also constitute the secretome of *A. tubingensis*. Although of unknown function, these proteins, which contain the signaling sequences at the N-terminal, are always secreted, and their probable functions are associated with metabolic supplies.

## Conclusions

Silver nanoparticles were biosynthesized using the secreted proteins from the fungus *A. tubingensis*. This fungal filtrate in contact with AgNO_3_ produced within 72 h AgNPs with 264.9 ± 3.2 nm in the hydrodynamic diameter, 35 ± 10 nm in the nanoparticle diameter and with a zeta potential of + 8.48 ± 0.45 mV. The nanoparticle formation was followed by UV-Vis spectroscopy, and the increase in the intensity of the SPR band was observed during AgNPs synthesis. The presence of fungal proteins in the AgNPs dispersion was verified by all spectrometric and spectroscopic analyses used. The FTIR along with the Raman data enabled us to identify the amino I, II, and III bands of proteins adhered to AgNP surface. Proteins formed covalent bonds with atoms at the surface of AgNPs surface due to their cysteine residues (Ag–S bonds) most likely. Secondary and tertiary structure features of proteins were preserved even when they were chemically bound to Ag atoms at the surface of the NPs. Eight proteins from *A. tubingensis* secretome were identified by MS/MS. All data collected and analyzed strongly indicate that not all fungal proteins bind to the formed AgNPs. However, some proteins enable the synthesis of AgNPs and provide stability to the formed nanosilver, not only through covalent bonds, but also due to attraction of other proteins through hydrogen bonds, electrostatic, or other supramolecular interactions, forming a multilayer, as evidenced by zeta potential measurements and size determinations of the AgNPs.

## Abbreviations

ADD, acquisition-dependent data; Ag, silver; AgNP, silver nanoparticles; DLS, dynamic light scattering; FF, fungal filtrate; FTIR, Fourier transform infrared spectroscopy; QTOF, quadrupole time-of-flight; TEM, transmission electron microscopy; UPLC, ultra performance liquid chromatography.

## Availability of Data and Materials

Mass spectrometry data treatments for the deconvolutions of raw spectra were performed with Transform software (Micromass, UK). MASCOT v.2.2 system (Matrix Science Ltd. http://www.matrixscience.com) and the data bank (UniProt http://www.uniprot.org/) searches were done in order to identify fungal proteins.

## Additional File

Additional file 1: Table S1.
*Aspergillus tubingensis* identified protein in the silver nanoparticles (AgNP) capping using LC-MS/MS. (DOC 58 kb)
