# Supplementary material for: Elucidating Protein Involvement in the Stabilization of the Biogenic Silver Nanoparticles
Source: Nanoscale Res Lett. 2016 Jun 29;11:313. doi: 10.1186/s11671-016-1538-y (PMC4927534; doi:10.1186/s11671-016-1538-y)
Supplement: Additional file 1: Table S1. — Aspergillus tubingensis identified protein in the silver nanoparticles (AgNP) capping using LC-MS/MS. (DOC 58 kb) [file 11671_2016_1538_MOESM1_ESM.doc]

**Elucidating protein involvement in the stabilization of biogenic silver nanoparticles**

Daniela Ballottin, Stephanie Fulaz, Michele L. Souza, Paola Corio, Alexandre G. Rodrigues, Ana O. Souza, Priscyla M. Gaspari, Alexandre F. Gomes, Fábio Gozzo, Ljubica Tasic.

***Table S1:*** *Aspergillus tubingensis* identified protein in the silver nanoparticles (AgNP) capping using LC-MS/MS:

| **Identified protein** | **pI** | **Molecular weight / kDa** | ***Score*** | **Identified peptides** | **% Cover** | **Function / Species with high homology** |
| --- | --- | --- | --- | --- | --- | --- |
| **Hypothetical protein An01g11010** | 4.06 | 39.0 | 93 | TLAYSDAQS GTRYPQTPMR | 4 | **Glycosidase** */ Aspergillus niger* |
| **Preproglucoamylase G2** | 4.41 | 57.0 | 61 | SIYTLNDGLS DSEAVAVGR | 3 | **Glucoamylase** /*Aspergillus niger* |
| **Hypothetical protein An04g08730** | 5.08 | 58.9 | 88 | TFVSNAV EQVIDDVTSR | 7 | **Unknown function** / *Aspergillus niger*, *Aspergillus kawachii* |
|  |  |  |  | NAFPNTLDT TIR |  |  |
|  |  |  |  | GEN LEQAVR |  |  |
| **Acid phosphatase** | 4.57 | 52.7 | 85 | YGHLWDGETVVPFFSSGYGR | 8 | **Acid phosphatase** / *Aspergillus niger* |
|  |  |  |  | VAFGNPYSIGNIVPMGGHLTIER |  |  |
| **Hypothetical protein An01g11010** | 4.06 | 39.8 | 73 | TLAYSDAQS GTRYPQTPMR | 4 | **Glycosidase** / *Aspergillus niger* |
| **Glucoamylase G1** | 4.19 | 65.4 | 66 | TLVDLFR | 14 | **Glucoamylase** / *Aspergillus niger*, *Aspergillus awamori* |
|  |  |  |  | FNVD ETAYTGSWGR PQR |  |  |
|  |  |  |  | ALANHKEVVDSFRSIYTLNDGLSDSEAVAVGR |  |  |
|  |  |  |  | DLTWSYAALLTANNRR |  |  |
|  |  |  |  | IESDDSVEWESDPNR |  |  |
| **Hypothetical protein An03g05200*** | 4.57 | 62.5 | 65 | ALQGFMGAFPQYSR | 7 | **Serine carboxypeptidase*** / *Aspergillus niger* |
|  |  |  |  | QAGQFAFVRVYESGHEVPFYQPLLALEMFER |  |  |
| **Hypothetical protein An09g00670** | 4.06 | 56.7 | 53 | DLPYLQELNTNVVR | 7 | **Glucanosyltransferase** / *Aspergillus niger* |
|  |  |  |  | SIGVGYSAADVSEIR |  |  |
|  |  |  |  | SFSNVPVMYGPK |  |  |
